# Phase 2 Open-Label Trial Investigating Percutaneous Laser Ablation for Treatment of Early-Stage Breast Cancer: MRI, Pathology, and Outcome Correlations

**DOI:** 10.1245/s10434-018-6623-2

**Published:** 2018-07-09

**Authors:** Barbara Schwartzberg, John Lewin, Osama Abdelatif, Jacqueline Bernard, Hanadi Bu-Ali, Simon Cawthorn, Margaret Chen-Seetoo, Sheldon Feldman, Sasirekha Govindarajulu, Lyn Jones, Arne Juette, Sanjay Kavia, Robert Maganini, Simon Pain, Mike Shere, Craig Shriver, Simon Smith, Alexandra Valencia, Eric Whitacre, Roger Whitney

**Affiliations:** 1 Sarah Cannon Research Institute at Rose Medical Center, Denver, CO USA; 2 Women’s Imaging Center, Denver, CO USA; 3 Tucson Pathology Associates, Tucson, AZ USA; 4 Walter Reed National Military Medical Center, Bethesda, MD USA; 5 Virginia Piper Cancer Institute, St. Paul, MN USA; 6 Southmead Hospital, Bristol, UK; 7 Herbert Irving Pavilion, Columbia University, New York, NY USA; 8 Montefiore Einstein Center for Cancer Care, Bronx, NY USA; 9 Norfolk and Norwich University Hospital, Norwich, UK; 10 Broomfield Hospital, Chelmsford, UK; 11 St. Alexius Breast Care Center, Bartlett, IL USA; 12 Breast Center of Southern Arizona, Tucson, AZ USA

## Abstract

**Background:**

An institutional review board-approved, multicenter clinical trial was designed to determine the efficacy and outcome of percutaneous laser ablation (PLA) in the treatment of invasive ductal breast carcinoma (IDC). Post-ablation magnetic resonance imaging (MRI) was compared with surgical pathology in evaluation of residual post-ablation IDC and ductal carcinoma in situ.

**Methods:**

Patients with a single focus of IDC 20 mm or smaller by pre-ablation MRI were treated with PLA. The patients underwent a 28-day post-ablation MRI, followed by surgical resection. Cell viability criteria were applied to pre- and post-ablation pathology specimens, which evaluated hematoxylin–eosin (H&E), cytokeratin (CK) 8/18, estrogen receptor, and Ki67 staining patterns.

**Results:**

In this study, 61 patients were reported as the intention-to-treat cohort for determination of PLA efficacy. Of these 61 patients, 51 (84%) had complete tumor ablation confirmed by pathology analysis. One subject’s MRI imaging was not performed per protocol, which left 60 subjects evaluable for MRI pathology correlation. Five patients (8.3%) had residual IDC shown by both MRI and pathology. Post-ablation discordance was noted between MRI and pathology, with four patients (6.7%) false-positive and four patients (6.7%) false-negative. The negative predictive value (NPV) of MRI for all the patients was 92.2% (95% confidence interval [CI], 71.9–91.9%). Of the 47 patients (97.9%) with tumors 15 mm or smaller, 46 were completely ablated, with an MRI NPV of 97.7% (95% CI, 86.2–99.9%).

**Conclusions:**

Percutaneous laser ablation is a potential alternative to surgery for treatment of early-stage IDC. Strong correlations exist between post-ablation MRI and pathologic alterations in CK8/18, ER, and Ki67 staining.

Percutaneous laser ablation (PLA) is a potential minimally invasive alternative to surgery in the treatment of early-stage breast cancer. This procedure is achieved by image-guided laser fiber insertion into the targeted cancer, after which the tumor is heated to a prespecified temperature (Figs. 1 and 2).

**Fig. 1:**
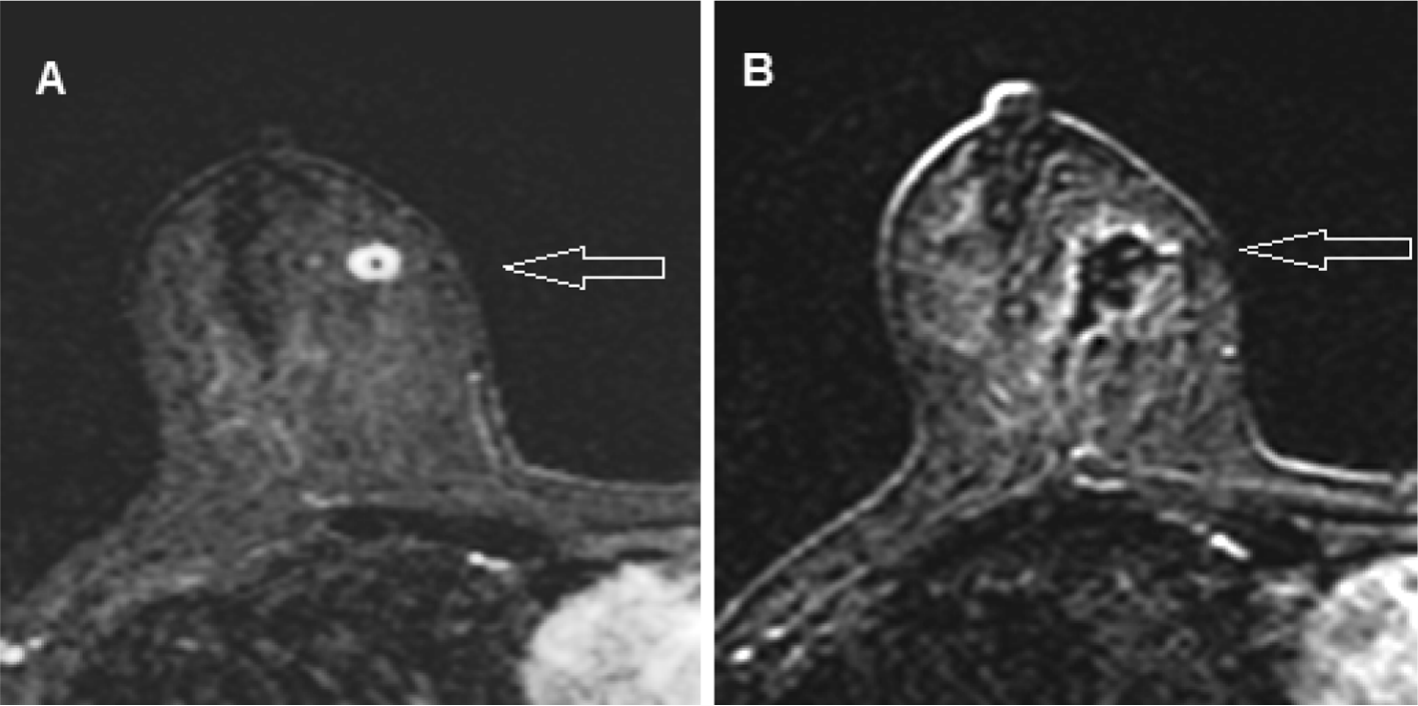
Example of magnetic resonance imaging (MRI). **a** Coronal gadolinium-enhanced, fat-suppressed, pre-ablation MRI image of an 11-mm grade 1 invasive ductal breast carcinoma (IDC). **b** Coronal MRI obtained 28 days after PLA showing ablation zone with no enhancement at the site of the cancer

**Fig. 2:**
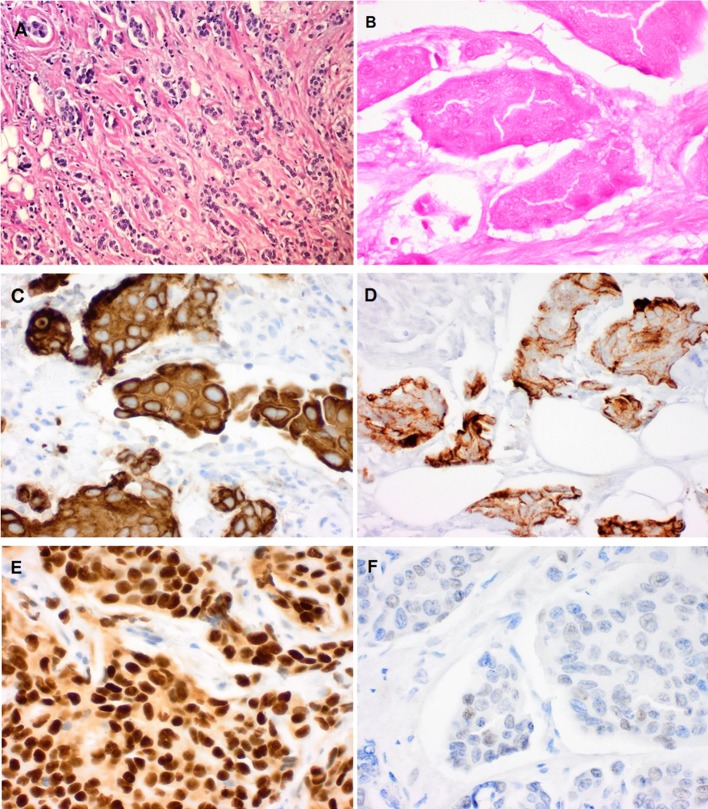
Pre- and post-ablation changes in pathology. All micrographs are × 40 magnification. **a** Hematoxylin and eosin (H&E) pre-ablation showing viable tumor cells. **b** H&E post-ablation showing shadows of nonviable tumor cells. **c** Pre-ablation cytokeratin 8/18 stains showing intact intensely stained tumor cells. **d** Post-ablation cytokeratin 8/18 stains showing low-intensity staining of tumor cells with incomplete membranous and cytoplasmic staining. **e** Pre-ablation estrogen receptor stains showing 3 + nuclear staining. **f** Post-ablation estrogen receptor stains showing complete loss of estrogen receptor nuclear staining

Laser ablation causes tumor destruction by conversion of light into thermal energy, creating direct and indirect damage to surrounding tissue. Direct heat injury occurs during heat deposition. Indirect heat injury occurs after thermal ablation, with production of progressive tissue damage involving tissue vaporization, microvascular damage, tissue necrosis, and immune cell activation. Temperature probes monitor tissue temperature changes, making PLA independent of breast tissue density, vascularity, procedural imaging changes, treatment time, energy delivered, and anesthetic injection.

Interstitial laser photocoagulation was reported in 1983 as a minimally invasive therapy for localized solid organ tumor destruction.^1^ Successful refinement of laser diode technology^2^ combined with image-guided laser probe placement in breast cancer patients,^3^ use of post-ablation MRI to determine breast cancer treatment response,^4^ and a better understanding of post-ablation pathology^5^ has provided significant improvements in this technique. The largest single-institution series (56 patients with malignant breast tumors) was reported in 2002.^6^ The overall success rate was 70%. This trial used an early version of the Novilase laser (Novian Health, Chicago, IL, USA) and included a learning phase plus technical and procedural changes.

The primary goal of this research study was to evaluate the performance of a newly improved percutaneous laser ablation device in achieving complete pathologic tumor ablation in a multicenter study. A secondary aim was to measure the performance of imaging in evaluation of complete tumor ablation. Tissue pathology at surgical excision was used as the gold standard.

## Methods

### Study Design

An open-label, phase 2 institutional review board (IRB)-approved, multicenter clinical trial was designed to determine the efficacy and outcome of PLA using Novilase Laser Therapy (Novian Health, Chicago, IL, USA), an 805-nm nominal wavelength laser diode source and system, in the treatment of invasive ductal breast carcinoma (IDC). Approved surgeons and radiologists experienced in image-guided breast procedures at nine sites enrolled subjects under IRB and Ethics Committee approved protocols. Secondarily, contrast-enhanced magnetic resonance imaging (MRI), mammography, and ultrasound imaging were evaluated as alternatives to surgical pathology in evaluation of residual post-PLA IDC and ductal carcinoma in situ (DCIS).

### Patient Eligibility Criteria

Women 18 to 80 years of age with a single focus of percutaneous biopsy-proven IDC measuring 20 mm or smaller were eligible for enrollment in the study. The cancer had to be visible by mammography, ultrasound, or both as a mass 20 mm or smaller or as a single cluster of microcalcifications 10 mm or smaller. The lesion had to be 5 mm or further from the skin and chest wall. Any intraductal component could not exceed 25%.

Before surgery, patients had to be willing to complete two European Organization for Research and Treatment of Cancer–Breast Cancer-Specific Quality-of-Life Questionnaires^7^ during the baseline visit and 28 days after PLA. The European Organisation for Research and Treatment of Cancer (EORTC) QLQ-BR23, a 23-question assessment, evaluated the patient’s perception of function and symptomatic treatment-associated side effects during their breast cancer treatment. The EORTC QLQ-C30, a generalized 30-question assessment, evaluated functional status during cancer treatment. Both questionnaires had a multiple-choice “not at all” to “very much” graded scale format. Trial score results were compared with the standardized EORTC reference means for early-stage breast cancer treatment. A score higher than the mean demonstrated an improved quality-of-life change in the functional scores. A score lower than the mean indicated fewer symptoms in the treatment-associated side effect scores.

Patients were excluded from the study if they were BRCA gene-positive, pregnant, or breast feeding. Patients were not considered if they had received neoadjuvant breast cancer therapy, had a history of PLA breast cancer treatment, or had recurrent breast cancer. Patients with benign tumors or DCIS were excluded. Additional exclusion criteria were morbid obesity, renal insufficiency, or comorbidities affecting life expectancy. Patients with cardiac pacemakers or metallic implants that would prevent safe MRI evaluation were not eligible for the study. Patients meeting the inclusion criteria were asked to enroll in this IRB-approved clinical trial and sign the informed consent.

### Imaging

Baseline imaging (mammography, ultrasound, contrast-enhanced MRI) was performed within 28 days before the PLA procedure. The same imaging was repeated within 28 days after the PLA procedure to evaluate for residual tumor. The 28-day timing of post-ablation imaging assessment and surgery was dictated by regulatory authority.

Mammography comprised a minimum of two standard views and two spot-compression magnification views. Ultrasound imaging consisted of gray-scale and color Doppler imaging, obtained in orthogonal planes. Contrast-enhanced MRI, performed with the patient in the prone position using a dedicated breast coil, consisted of T1- and T2-weighted pre-contrast imaging followed by T1-weighted dynamic imaging performed before and serially after administration of a weight-based dose of a standard gadolinium contrast agent. A minimum of five post-contrast time points were obtained.

Interpretation was performed by both the site radiologist (for clinical use) and a central group of study radiologists. Baseline imaging was used to determine study eligibility based on tumor size and distance from the skin and chest wall. Post-treatment imaging was used to evaluate ablation completeness. The baseline imaging was available for use in interpretation of the post-treatment imaging. Evaluation of residual tumor by MRI was based primarily on the presence and nature of contrast enhancement in the region of the original tumor. Ultrasound imaging, including color Doppler, was repeated at the time of PLA for those lesions being ablated under ultrasound guidance.

### Procedure

The patients wore protective goggles during their treatment in the breast center procedure rooms under local anesthesia with ultrasound or stereotactic image-guided PLA. A 14-gauge laser probe, which contained a thermal sensor at the tip, was placed under image guidance into the targeted cancer center. The laser fiber was passed through the probe, and the laser tip was placed 1 to 2 mm beyond the probe end. An infusion pump attached to the laser probe allowed for sterile saline infusion at a rate of 0.4 to 1 ml/min continuous flow along the side of the laser fiber. A thermal probe, which contained five temperature sensors spaced 5 mm apart, was placed parallel to the laser probe via a probe guide. This placed the thermal probe at a distance of 1 to 2 cm from the desired tumor ablation zone. The thermal probe allowed real-time monitoring of tissue temperatures at the periphery of the lesion.

Treatment was terminated when all peripheral temperature sensors reached 60 °C or if peripheral temperatures were maintained at 51 °C or higher for at least 2 min. Saline flow rate adjustments and coolant spray applications were performed as needed to mitigate pain and protect against skin burn. Pain levels were assessed using a pain scale of 0 to 10 throughout the procedure.

The patients were evaluated within 28 days post-ablation using mammogram, ultrasound, and MRI, after which they underwent surgical excision. Surgical resection together with sentinel lymph node biopsy, if appropriate, was performed within 28 days after the PLA procedure.

Adverse events were documented using International Council for Harmonization of Technical Requirement (ICH) for Good Clinical Practices guidelines 1.2.^8^ Mild events were defined as events easily tolerated by the patient, which caused minimal discomfort and did not interfere with everyday activities. Moderate events were those that were sufficiently discomforting to interfere with normal everyday activities, whereas severe adverse events were those that prevented normal everyday activities. The patients were offered standard-of-care adjuvant therapy and followed for 5 years at designated intervals.

### Pathology Analysis

Pre-ablation pathology was validated by needle core biopsy. After ablation, the entire surgically excised tissue specimen was assessed by protocol-trained institutional breast pathologists who used post-ablation pathology protocol guidelines for gross and microscopic pathology analysis. Central pathology review of representative slides from all 61 cases was performed by a single pathologist (O.A.). Cell viability criteria were applied to pathology specimens during central pathology laboratory review using pre- and post-ablation hematoxylin and eosin (H&E), CK8/18, ER, and Ki67 staining.

### Data Collection and Statistical Analysis

Patient imaging and pathology results, evaluated locally and by central independent core radiology and pathology review, were collected in a central repository. A primary end-point statistical analysis was performed using the SAS PROC GLIMMIX program (SAS Institute, Inc., Cary, NC, USA) and Mood’s over-dispersion median statistical analysis, which accounted for multi-institutional differences. Summary statistics were generated using a generalized linear model analysis.

## Results

For this study, 61 women treated with PLA from June 2012 to May 2015 (Table 1) met the primary end point as the intention-to-treat (ITT) cohort determining PLA efficacy. Post-ablation MRI imaging of one subject violated protocol, which left 60 patients for MRI pathology correlation.

**Table 1:** Patient and tumor characteristics

Number of women	61
Mean age: years (range)	64 (42–77)
Race	
Caucasian	52
African American	3
Hispanic/Latino	5
Asian	1
Mean tumor size (pre-ablation MRI): mm (range)	11.3 (4.0–19.0)
Histology	
Infiltrating ductal	47
Infiltrating ductal/ductal carcinoma in situ	9
Ductal carcinoma in situ	1
Other	3
Tumor grade	
1	24
2	31
3	6
Lymphovascular invasion	3
Molecular subtype	
HER2–/estrogen receptor+	50
HER2 +/estrogen receptor+	4
HER2 +/estrogen receptor–	2
HER2 equivocal	2
HER2 not done	2
HER2–/estrogen receptor–	1

*HER2* human epidermal growth factor receptor 2

### PLA Procedure

All 61 enrolled subjects were treated with PLA. Of these 61 patients, 60 underwent PLA using ultrasound guidance, with 1 patient using stereotaxis. The mean laser time was 15.8 min (range, 14.5–36.5 min). The mean energy expenditure was 4560 J (range, 864–11,231 J). The mean saline infusion was 8.7 ml (range, 2.5–21.0 ml), and the mean local anesthesia injection was 12.3 ml (range, 2–50 ml). The total procedure time averaged less than 1 h. All 61 patients underwent surgical excision of the ablated areas, with 55 patients undergoing lumpectomy and 6 patients undergoing mastectomy.

### Adverse Events

No serious adverse events occurred. Eight adverse events considered mild by the investigators included lump (1 patient), blister (2 patients), hematoma (1 patient), erythema (1 patient), and fat necrosis (3 patients). Six adverse events considered moderate by the investigators included pain (4 patients), lump (1 patient), and seroma (1 patient). All adverse events resolved.

### Protocol Deviations

A total of 19 protocol deviations were documented in 17 of the 61 PLA cases. Documentation for 12 procedural protocol deviations showed that target temperatures were not reached, whereas documentation for 4 procedural protocol deviations showed tumor mistargeting. Three protocol deviations documented screening failures as PLA was performed on tumors larger than protocol specifications. All protocol guidelines were fulfilled in the PLA procedures performed for 44 of the 61 subjects. Of these 44 patients, 40 (91%) had complete pathologic ablation.

### Learning Curve

A steep first-case learning curve was encountered at each site. Only two of the first cases at the nine sites achieved technical successes. After these 9 first cases, 42 (81%) of the remaining 52 ITT cases were deemed technical successes, with 5 (9.6%) of these 52 cases found to have post-PLA macroscopic residual tumor.

### Patient Experience

The patients reported qualitative satisfaction with the procedure. The average maximum pain reported by the 61 patients during treatment was 4.2 ± 2.9 (range 0–10). Return to activities of daily living averaged less than 8 h and was immediate for 45 (78%) of 58 subjects. At the 28-day follow-up evaluation, 97% of the 58 subjects who responded to cosmesis questions rated their cosmetic satisfaction as excellent (64%) or good (33%). One subject reported a significant scar at the 28-day follow-up evaluation.

Health-related quality-of-life assessments using the EORTC QLQ-BR23 and QLQ-C30 surveys were completed by all 61 subjects during their baseline visit and at their 28-day follow-up evaluation. Compared with the EORTC reference mean for early-stage breast cancer treatment, a positive meaningful change of at least 5 points was reported by the 61 subjects in this trial 28 days after ablation for all functional scales. This indicated an improved outlook and function after PLA treatment. The scores reflecting severity of treatment-associated side effects reported in this trial were less than published post-lumpectomy EORTC data or the EORTC reference mean.^9^ In particular, scores were lower than the reference mean for published lumpectomy data^9^ for fatigue (− 17.49), pain (− 13.40), and insomnia (− 15.41). This indicated that PLA treatment-associated side effects were less severe.

### Pathology Analysis

All 61 ITT cases demonstrated characteristic post-ablation gross pathology changes. A series of concentric rings surrounded the central area of char, which housed the laser tip. This was immediately surrounded by an area of gray coagulation followed by a ring of hyperemia. For evaluation of post-ablation changes, CK8/18 and ER microscopic stains were superior to H&E and Ki67 in documenting post-ablation cell viability. The CK8/18 stain demonstrated post-ablation tumor destruction by staining either totally negative or by having a granular, interrupted membranous staining pattern. In addition, CK8/18 staining changed from a cellular to a “stringy” pattern, which documented cellular disruption.

Of the 61 cancers in this study, 58 (95%) were ER-positive. Lack of post-ablation ER staining in these specimens confirmed non-viability of residual tumor.

Pathologic complete primary tumor ablation was achieved in 51 (84%) of the 61 ITT cases. The volume of the primary tumor was reduced 96%. Three procedures had less than 0.6% cancer cells in any cm^2^ section of the target tumors. These cells, which represented a minute amount of microscopic residual tumor, were scattered, noncontiguous, and too small to be seen by post-ablation MRI.

Residual tumor was present at the index targeted tumor site in 10 (16%) of the 61 ITT lesions. The overall success rate for ablation of the index tumors was 84%. One patient was mistargeted, which represented a 100% post-PLA residual volume. Residual tumor averaged 12.1% (range, 0.3–40%) in the remaining nine ITT subjects. Two additional subjects had residual tumor represented as previously undiagnosed secondary tumors, which denoted multifocal disease.

### Post-Ablation Imaging and Pathology Correlation

Radiology-pathology correlations were performed for the 60 cases in which MRI images were performed per protocol. These were categorized into four groups. “True-positive” described a group in which both imaging and post-excision pathology showed macroscopic residual tumor, whereas “true-negative” described a group in which neither imaging nor post-excision pathology showed macroscopic residual tumor.

“False-negative” constituted a group in which imaging showed no macroscopic residual tumor whereas post-excision pathology demonstrated this. “False-positive” represented a group in which imaging showed macroscopic residual tumor whereas post-excision pathology did not. The results were summarized for the 60 patients with images performed per protocol (Table 2). The MRI-negative predictive value (NPV), specificity, sensitivity, and accuracy were superior to mammography and ultrasound in this study.

**Table 2:** Negative predictive value (NPV), specificity, sensitivity, and accuracy of imaging methods in detection of residual breast cancer 28 days after PLA

	MRI (%)	Ultrasound (%)	Mammogram (%)
NPV	92	89	75
Specificity	92	67	33
Sensitivity	64	67	58
Accuracy	87	67	39

*MRI* magnetic resonance imaging

Correlations with pathology were performed for the 60 patients whose MRI images were obtained per protocol (Table 3). The NPV of MRI for these 60 patients was 92.2% (95% confidence interval [CI], 71.9–91.9%). The NPV of MRI for the 47 patients with cancers 15 mm or smaller was 97.7% (95% CI, 86.2–99.9%).

**Table 3:** Post-ablation MRI and surgical pathology correlation in detection of residual breast cancer 28 days after percutaneous laser ablation (PLA)

		Residual *n* (%)	No residual *n* (%)
Pathology results	MRI (all cancers)^a^		
	Residual	5 (8.3) True-positive	4 (6.7) False-negative
	No residual	4 (6.7) False-positive	47 (78.3) True-negative
	MRI results for cancers ≤ 15 mm		
	Residual	0 (0) True-positive	1 (2.1) False-negative
	No residual	3 (6.4) False-positive	43 (91.5) True-negative

*MRI* magnetic resonance imaging

^a^60 subjects were evaluable by both MRI and post-PLA pathology

### Patient Follow-Up Evaluation

Two recurrences were reported during a mean follow-up period of 43 months (range, 34–65 months). Both patients had complete ablations documented by post-ablation MRI and pathology. One patient, with an 8.5-mm ER-positive tumor, declined any additional treatment after PLA and excision. At 3 years, a recurrence was documented 25 mm from the PLA site. A second patient, with an 11.5-mm triple-negative tumor, was treated with chemotherapy plus whole-breast radiation after PLA and resection. A skin recurrence was noted in the lumpectomy incision at the 3-year follow-up assessment. No patient experienced PLA-associated sequelae.

## Discussion

Percutaneous laser ablation has gained a potential place in the paradigm of nonsurgical treatment for early-stage breast cancer.^10^ Multiple single-institution reports have documented the success of PLA.^3^,^4^,^6^ Our trial was designed to evaluate feasibility and outcomes with a newly improved laser device in a multi-institutional setting. This study documented complete ablation, defined as no residual tumor in the post-ablation excised pathologic specimens, in 84% of 61 ITT cases and in 91% of 44 cases that met the inclusion, targeting, and temperature protocol criteria. These outcomes reflected findings within the 28-day post-ablation window dictated by regulatory authority. These results could possibly be improved if given additional post-ablation time due to continued progression of tumor necrosis and cancer cell death.

Stereotactic localization was combined with an early version of the Novilase laser during PLA procedures in a single-institution series of 56 patients, which reported a 70% PLA success rate.^6^ Ablation failures in that single-institution series were due to inadequate visualization after injection of local anesthesia and technical device issues. Use of ultrasound imaging and refinements in the Novilase laser device resulted in improved outcomes in our series. Investigator experience evolved during the course of our trial, although center-to-center variability was found to be minimal and reproducible across sites. Suboptimal laser probe placement during early cases resulted in incomplete ablation. Later approaches emphasized probe placement through the longest axis of the tumor. The full range of power available in the laser unit was not used in many cases, which led to longer treatment times and increased amounts of local anesthesia. Excess local anesthesia acted as a heat sink, which negated reaching target temperatures. Modifications in investigator training and the *User Manual* emphasized these improvements in proper protocol procedure, with the expectation of improved outcomes.

For documention of post-ablation success, MRI was found to be a useful tool in 60 cases that had MRI imaging performed per protocol. The NPV of MRI was 92% in IDC 20 mm or smaller and 97.7% in IDC 15 mm or smaller. Changes in MRI occurring at longer post-ablation intervals were not established. This could be the source of future trials.

Positive patient experience has been an important component in assessment of new technologies used in the treatment of breast cancer.^11^ In the current trial, PLA-associated adverse events were minimal. Good to excellent patient satisfaction was reported by 56 (96.6%) of 58 patients 28 days after PLA. The patients reported higher, more positive functional and lower, less severe symptomatic scores than the mean for breast surgery patients who completed the EORTC QLQ-BR23 and QLQ-C30 questionnaires.

The two (3.3%) post-PLA plus resection recurrences documented in a mean follow-up assessment at 43 months did not reflect consequences associated with PLA. No PLA-associated sequelae occurred.

## Conclusions

Percutaneous laser therapy, which works by focal destruction, is a potential alternative to traditional breast cancer conservation surgery for treatment of early-stage IDC. Strong correlations exist between post-ablation MRI findings and pathologic alterations in CK8/18, ER, and Ki67 staining. Clinical trials that evaluate PLA efficacy and outcome in the absence of subsequent surgical resection are necessary to further determine the potential of this breast cancer therapy.
